# Supplementary material for: Ubiquitination-mediated Golgi-to-endosome sorting determines the toxin-antidote duality of fission yeast wtf meiotic drivers
Source: Nat Commun. 2023 Dec 14;14:8334. doi: 10.1038/s41467-023-44151-9 (PMC10721834; doi:10.1038/s41467-023-44151-9)
Supplement: Supplementary file 3 — Description of Additional Supplementary Files [file 41467_2023_44151_MOESM3_ESM.pdf]

Description of Additional Supplementary Files

File Name: Supplementary Data 1
Description: *S. pombe* proteins identified in the AP-MS experiment shown in Figure 2c.

File Name: Supplementary Data 2
Description: *S. pombe* and *S. cerevisiae* strains used in this study.

File Name: Supplementary Data 3
Description: plasmids used in this study.
