# Supplementary material for: Ubiquitination-mediated Golgi-to-endosome sorting determines the toxin-antidote duality of fission yeast wtf meiotic drivers
Source: Nat Commun. 2023 Dec 14;14:8334. doi: 10.1038/s41467-023-44151-9 (PMC10721834; doi:10.1038/s41467-023-44151-9)
Supplement: Supplementary file 7 — Reporting Summary [file 41467_2023_44151_MOESM7_ESM.pdf]

nature portfolio

Corresponding author(s): Li-Lin Du

Last updated by author(s): Nov 19, 2023

Reporting Summary

Nature Portfolio wishes to improve the reproducibility of the work that we publish. This form provides structure for consistency and transparency in reporting. For further information on Nature Portfolio policies, see our Editorial Policies and the Editorial Policy Checklist.

Statistics

For all statistical analyses, confirm that the following items are present in the figure legend, table legend, main text, or Methods section.

n/a	Confirmed	
☐	☒	The exact sample size (*n*) for each experimental group/condition, given as a discrete number and unit of measurement
☒	☐	A statement on whether measurements were taken from distinct samples or whether the same sample was measured repeatedly
☒	☐	The statistical test(s) used AND whether they are one- or two-sided<br>*Only common tests should be described solely by name; describe more complex techniques in the Methods section.*
☒	☐	A description of all covariates tested
☒	☐	A description of any assumptions or corrections, such as tests of normality and adjustment for multiple comparisons
☒	☐	A full description of the statistical parameters including central tendency (e.g. means) or other basic estimates (e.g. regression coefficient) AND variation (e.g. standard deviation) or associated estimates of uncertainty (e.g. confidence intervals)
☒	☐	For null hypothesis testing, the test statistic (e.g. *F*, *t*, *r*) with confidence intervals, effect sizes, degrees of freedom and *P* value noted<br>*Give P values as exact values whenever suitable.*
☒	☐	For Bayesian analysis, information on the choice of priors and Markov chain Monte Carlo settings
☒	☐	For hierarchical and complex designs, identification of the appropriate level for tests and full reporting of outcomes
☒	☐	Estimates of effect sizes (e.g. Cohen's *d*, Pearson's *r*), indicating how they were calculated

Our web collection on statistics for biologists contains articles on many of the points above.

Software and code

Policy information about availability of computer code

Data collection	SoftWoRx (7.0.0) on a DeltaVision microscope system and Fusion (2.3.0.54) on a Dragonfly microscope system were used to acquire images.
Data analysis	SoftWoRx (7.0.0), Photoshop CS6 (13.0), and Fiji (2.9.0) were used to analyze images.<br>TOPCONS web server (https://topcons.cbr.su.se/) and PolyPhobius web server (http://phobius.sbc.su.se/poly.html) were used to predict the transmembrane topology of proteins.<br>MAFFT web server (https://mafft.cbrc.jp/alignment/server/) was used to generate sequence alignments.

For manuscripts utilizing custom algorithms or software that are central to the research but not yet described in published literature, software must be made available to editors and reviewers. We strongly encourage code deposition in a community repository (e.g. GitHub). See the Nature Portfolio guidelines for submitting code & software for further information.

Data

Policy information about availability of data

All manuscripts must include a data availability statement. This statement should provide the following information, where applicable:

- Accession codes, unique identifiers, or web links for publicly available datasets
- A description of any restrictions on data availability
- For clinical datasets or third party data, please ensure that the statement adheres to our policy

The authors declare that all data supporting the findings of this study are available within the paper and its supplementary information files. Source data are provided with this paper.

Research involving human participants, their data, or biological material

Policy information about studies with human participants or human data. See also policy information about sex, gender (identity/presentation), and sexual orientation and race, ethnicity and racism.

Reporting on sex and gender	N/A
Reporting on race, ethnicity, or other socially relevant groupings	N/A
Population characteristics	N/A
Recruitment	N/A
Ethics oversight	N/A

Note that full information on the approval of the study protocol must also be provided in the manuscript.

Field-specific reporting

Please select the one below that is the best fit for your research. If you are not sure, read the appropriate sections before making your selection.

☒ Life sciences ☐ Behavioural & social sciences ☐ Ecological, evolutionary & environmental sciences

For a reference copy of the document with all sections, see nature.com/documents/nr-reporting-summary-flat.pdf

Life sciences study design

All studies must disclose on these points even when the disclosure is negative.

Sample size	Sample size was not predetermined using statistical methods in this study. Instead, the sample sizes were determined based on community standards for relevant experiments. For instance, approximately 100 spores were placed onto plates for the tetrad analysis, while more than 100 cells were observed for microscopy analyses. These sample sizes are consistent with those used in published studies (PMID: 28631610; PMID: 36227631; PMID: 32735772).
Data exclusions	No data were excluded.
Replication	All experiments were repeated independently at least two times. For figures showing results from representative experiments (such as micrographs and blots), we state in figure legends how many times each experiment was repeated independently with similar results.
Randomization	In this study, randomization was not utilized since the experiments were conducted on uniform biological material, specifically yeast cells. As a result, randomization was deemed unnecessary for the purposes of this research.
Blinding	In this study, blinding was not used due to the fact that the experiments were designed and performed by individual investigators who were aware of the identities of the analyzed samples. Blinding is not typically employed in this field of research.

Reporting for specific materials, systems and methods

We require information from authors about some types of materials, experimental systems and methods used in many studies. Here, indicate whether each material, system or method listed is relevant to your study. If you are not sure if a list item applies to your research, read the appropriate section before selecting a response.

Materials & experimental systems

n/a	Involved in the study
☐	☒ Antibodies
☒	☐ Eukaryotic cell lines
☒	☐ Palaeontology and archaeology
☒	☐ Animals and other organisms
☒	☐ Clinical data
☒	☐ Dual use research of concern
☒	☐ Plants

Methods

n/a	Involved in the study
☒	☐ ChIP-seq
☒	☐ Flow cytometry
☒	☐ MRI-based neuroimaging

Antibodies

Antibodies used

Primary and secondary antibodies used in this study are all from commercial sources. They include:
1. Mouse monoclonal anti-GST, Beijing Protein Innovation, Cat#AbM59001-2H5-PU
2. Mouse monoclonal anti-FLAG, Sigma Aldrich, Cat#F3165
3. Mouse monoclonal anti-HA, MBL International, Cat#M180-3
4. Mouse monoclonal anti-myc, Huaxingbio, Cat#HX1802
5. Mouse monoclonal anti-GFP, Roche, Cat#11814460001
6. Rabbit polyclonal anti-mCherry, Abcam, Cat#ab167453
7. Goat anti-Mouse IgG, Sigma Aldrich, Cat#A4416
8. Goat anti-Rabbit IgG, Sigma Aldrich, Cat#A6154

Validation

Validation information for the primary antibodies used in this study is shown below.
1. Mouse monoclonal anti-GST, Beijing Protein Innovation, Cat#AbM59001-2H5-PU
http://www.proteomics.org.cn/product/185.html
This antibody was used for immunoblotting in Li et al. Dev Cell 2017 (PMID: 29056553).
2. Mouse monoclonal anti-FLAG, Sigma Aldrich, Cat#F3165
https://www.sigmaaldrich.cn/CN/en/product/sigma/f3165
Google Scholar search showed that this antibody has been used in thousands of publications.
One of the earliest publications using this antibody is Zhang et al. Proc Natl Acad Sci U S A 1999 (PMID: 10411939), which used this antibody for immunoblotting.
3. Mouse monoclonal anti-HA, MBL International, Cat#M180-3
https://www.mblintl.com/products/m180-3/
Google Scholar search showed that this antibody has been used in hundreds of publications.
One of the earliest publications using this antibody is Iguchi et al. J Biol Chem 2013 (PMID: 23754282), which used this antibody for immunoblotting.
4. Mouse monoclonal anti-myc, Huaxingbio, Cat#HX1802
http://www.huaxingbio.com/pd.jsp?id=333&nSL=%5B5%2C6%2C7%5D#skeyword=HX1802&_pp=0_35
This antibody was used for immunoblotting in Ji et al. PLoS Pathog 2019 (PMID: 31216343).
5. Mouse monoclonal anti-GFP, Roche, Cat#11814460001
https://www.sigmaaldrich.cn/CN/en/product/roche/11814460001
Google Scholar search showed that this antibody has been used in thousands of publications.
One of the earliest publications using this antibody is Arts et al. Nat Genet 2007 (PMID: 17558407), which used this antibody for immunoblotting.
6. Rabbit polyclonal anti-mCherry, Abcam, Cat#ab167453
https://www.abcam.com/products/primary-antibodies/mcherry-antibody-ab167453.html
The above web page lists 392 publications using this antibody. One of the earliest publications using this antibody is Lee et al. Proc Natl Acad Sci U S A 2014 (PMID: 24550500), which used this antibody for immunoblotting.
